# Permanent stoma rate and long-term stoma complications in laparoscopic, robot-assisted, and transanal total mesorectal excisions: a retrospective cohort study

**DOI:** 10.1007/s00464-023-10517-9

**Published:** 2023-11-06

**Authors:** T. A. Burghgraef, R. T. J. Geitenbeek, M. Broekman, J. C. Hol, R. Hompes, E. C. J. Consten, The MIRECA Study Group

**Affiliations:** 1 Department of Surgery, Meander Medical Center, Maatweg 3, 3813 TZ Amersfoort, The Netherlands; 2 Department of Surgery, University Medical Center Groningen, Hanzeplein 1, 9713 GZ Groningen, The Netherlands; 3 Department of Surgery, Amsterdam UMC, VUmc, Amsterdam, The Netherlands; 4 Department of Surgery, Amsterdam UMC, AMC, Amsterdam, The Netherlands

**Keywords:** Rectal cancer, Permanent stoma, Minimally invasive, Total mesorectal excision

## Abstract

**Background:**

The surgical resection of rectal carcinoma is associated with a high risk of permanent stoma rate. Primary anastomosis rate is suggested to be higher in robot-assisted and transanal total mesorectal excision, but permanent stoma rate is unknown.

**Methods:**

Patients undergoing total mesorectal excision for MRI-defined rectal cancer between 2015 and 2017 in 11 centers highly experienced in laparoscopic, robot-assisted or transanal total mesorectal excision were included in this retrospective study. Permanent stoma rate, stoma-related complications, readmissions, and reoperations were registered. A multivariable regression analysis was performed for permanent stoma rate, stoma-related complications, and stoma-related reoperations.

**Results:**

In total, 1198 patients were included. Permanent stoma rate after low anterior resection (with anastomosis or with an end colostomy) was 40.1% in patients undergoing laparoscopic surgery, 21.3% in patients undergoing robot-assisted surgery, and 25.6% in patients undergoing transanal surgery (*P* < 0.001). Permanent stoma rate after low anterior resection with an anastomosis was 17.3%, 11.8%, and 15.1%, respectively. The robot-assisted and transanal techniques were independently associated with a reduction in permanent stoma rate in patients who underwent a low anterior resection (with anastomosis or with an end colostomy) (OR 0.39 [95% CI 0.25, 0.59] and OR 0.35 [95% CI 0.22, 0.55]), while this was not seen in patients who underwent a restorative low anterior resection. 45.4% of the patients who had a stoma experienced stoma-related complications, 4.0% were at least once readmitted, and 8.9% underwent at least one reoperation.

**Conclusions:**

The robot-assisted and transanal techniques are associated with a lower permanent stoma rate in patients who underwent a low anterior resection.

**Supplementary Information:**

The online version contains supplementary material available at 10.1007/s00464-023-10517-9.

Total mesorectal excision (TME) is the standard treatment for rectal cancer. It can be performed using open, laparoscopic (L-TME), robot-assisted (R-TME), and transanal TME (TaTME) [1], and is associated with a high incidence of stoma construction [2, 3].

Based on tumor characteristics and patient preferences, a low anterior resection (LAR) with or without the construction of an anastomosis, or an abdominoperineal resection (APR) may be performed. In patients undergoing an APR, and patients undergoing a LAR without anastomosis, an end colostomy is constructed. In patients undergoing a LAR with anastomosis, a temporary stoma may be considered, although the (dis)advantages are strongly debated [4–6]. While most diverting stomas and some end colostomies constructed during a LAR are intended to be reversed, up to 20% will never be reversed, and a considerable proportion of rectal cancer patients end up having a permanent stoma [7, 8].

Although it is unclear whether having a stoma influences quality of life, stoma-related complications are often described in patients having a stoma [9, 10]. As stoma-related complications are known to occur more frequently with an increased duration of the stoma, permanent stomas are suggested to have a high rate of stoma-related morbidity and might therefore result in a stronger decline in quality of life compared to patients having a temporary stoma [9–13]. Recent studies showed that R-TME and TaTME performed by experienced surgeons resulted in significantly more primary anastomoses, compared to L-TME [14–17]. These results suggest that R-TME and TaTME are better capable of constructing an anastomosis; however, it is unknown whether this effect will remain on the long term, and will result in a lower permanent stoma rate. Therefore, this study aims to compare L-TME, R-TME, and TaTME with regard to permanent stoma rate.

## Materials and methods

This is a retrospective cohort study of rectal cancer patients operated between January 1st, 2015 and December 31st, 2017 in eleven large Dutch teaching centers, performed by experienced rectal cancer surgeons. As it is difficult to reach the same levels of proficiency in all three techniques by the same surgeon, we compared the results of high-volume hospitals specialized predominantly in one of the three techniques. Centers were ‘dedicated’ in either L-TME, R-TME, or TaTME and only one of the techniques was the standard technique.

### Objectives

The primary objective was to determine the permanent stoma rate among all patients who underwent a LAR (with anastomosis or with an end colostomy). Secondary objectives included the permanent stoma rate among all L-TME, R-TME, and TaTME patients (this included LAR with colostomy, LAR with anastomosis and APR) and among all patients who underwent a restorative LAR (with the construction of an anastomosis). Other objectives included the determination of the incidence of stoma-related complications and reinterventions during long-term follow-up in all patients having a stoma.

### Patients

Patients were eligible for inclusion if they (1) underwent a TME because of primary rectal cancer according to the rectal cancer definition as proposed by d’Souza et al. [18], (2) were operated between January 1st, 2015 and December 31st, 2017, (3) were registered in the national prospective Dutch Colo-Rectal Audit (DCRA) database, and (4) underwent minimally invasive TME. Patients were excluded if (1) they already had a stoma unrelated to treatment of the rectal cancer at diagnosis or (2) when they underwent a TME with palliative intent or due to recurrent disease.

### Data

An already existing retrospective database aimed at comparing L-TME, R-TME, and TaTME was used [14]. In short, the database consisted of prospectively registered data from the DCRA, while missing data, and additional data not present in the DCRA database were added through the patients’ electronic medical record. Informed consent was waived by the regional medial ethical committee, and both the regional medical ethical committee and all local hospital ethical committees gave their approval for the study (MEC-U: AW19-023). The study design and drafting of the article were in accordance with the STROBE statement [19].

### Outcomes

A permanent stoma was defined as a stoma created during the initial surgery or later, that remained until the patient’s last follow-up visit or death of the patient. A primary stoma was defined as a stoma constructed prior or during the TME, a secondary stoma was defined as a stoma constructed after the TME, and a tertiary stoma was defined as a stoma constructed after initial stoma reversal of the primary or secondary stoma. Stoma-related complications included ileus due to the stoma, high-output stoma, stoma prolapse, parastomal hernia, stricture of the stoma, dehiscence of the stoma, necrosis of the stoma, parastomal skin complications, and infectious complications. Stoma-related complications were defined as short term if they occurred within the first 30 days after stoma construction, and as long term if they occurred more than 30 days after stoma construction. Furthermore, the following data related to the stoma were registered: type of stoma (diverting ileostomy, end ileostomy, diverting colostomy, end colostomy), timing of stoma construction (prior to initial resection, during initial resection, after surgical resection as a consequence of surgical complications, after reversal), reversal of stoma, time to reversal, readmissions related to the stoma, and reoperations related to the stoma. Readmissions and reoperations related to reversal were not registered.

Baseline characteristics were age, body mass index (BMI), sex, American Society of Anesthesiologists’ (ASA) classification, history of abdominal surgery, use of neoadjuvant therapy, TNM stage [21], and distance from the inferior border of the tumor from the anorectal junction. Furthermore, type of minimally invasive TME-technique, type of surgical procedure, surgical complications, reinterventions, and anastomotic leakage were registered. Anastomotic leakage was defined according to the ISREC criteria [20], and registered during the whole follow-up period. Type of minimally invasive TME-technique was defined as L-TME, R-TME, and TaTME. In case of TaTME, the abdominal part of the procedure was performed laparoscopically. Type of the surgical procedure was defined as an APR, a LAR or a restorative LAR. An APR was defined as either an intersphincteric, a classic or an extralevatoric APR with proctectomy. A LAR could be either with the construction of an anastomosis or without the construction of an anastomosis but with an end ostomy. Finally, the group patients who underwent a restorative LAR all had an anastomosis constructed during the initial resection.

### Statistical analysis

For the comparative analyses, outcomes of LAR (with anastomosis or with an end colostomy) were compared between the L-TME, R-TME, and TaTME technique. In addition, to account for selection bias regarding, outcomes were compared between all patients who underwent a L-TME, R-TME, or TaTME, thereby including the patients who underwent an APR. Furthermore, data were stratified per center (L-TME center, R-TME center and TaTME center) as well and added in the supplemental tables. The Fisher’s exact test was used for comparing categorical differences. The Wilcoxon-rank sum test was used for continuous variables that were not normally distributed data, whereas the student’s t test was used for normally distributed data.

To control for confounding factors, multivariable regression analyses were performed. Multivariable analyses were performed using backward logistic regression analysis for permanent stoma rate, stoma-related complications, and stoma-related reoperations. Variables included in the logistic regression analysis for permanent stoma rate of all TME patients (LAR with anastomosis or with an end colostomy, and APR) and for patients undergoing a LAR (with or without the construction of an anastomosis) were based on literature, and included age, gender, BMI, ASA classification, history of abdominal surgery, neoadjuvant therapy, cTNM stage, mesorectal fascia (MRF) involvement, tumor distance to the ARJ, type of minimally invasive TME-technique, and duration of follow-up. For the multivariable analysis of permanent stoma rate after restorative LAR (with the construction of an anastomosis), the following two variables were added as well: diverting stoma during primary resection and anastomotic leakage. For stoma complications and stoma interventions, the following risk factors were included: age, gender, BMI, ASA classification, history of abdominal surgery, neoadjuvant therapy, type of stoma, moment of stoma construction, and total duration of the stoma [22–29]. Missing data were imputed using multiple imputation if data were missing at random or if data were missing completely at random. Analyses were performed using’R’ version 4.1.3.

## Results

### Baseline characteristics

**Fig. 1:**
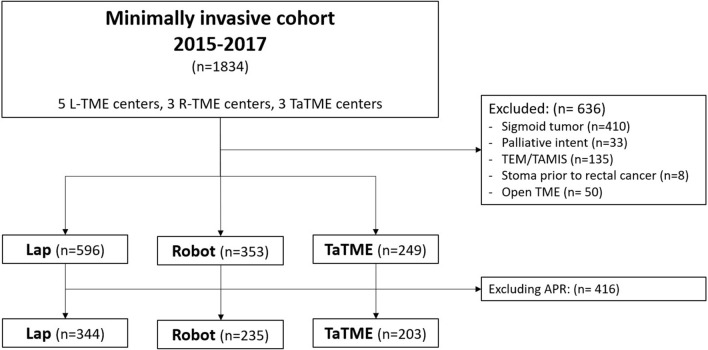
Flow diagram of included patients. N: number of patients, L-TME: laparoscopic total mesorectal excision, R-TME: robot-assisted total mesorectal excision, TaTME: transanal total mesorectal excision, TEM: transanal endoscopic microsurgery, TAMIS: transanal minimally invasive surgery, APR: abdominoperineal resection

In total, 1834 patients were identified in the retrospective cohort; 1198 patients were included in the analysis. A TME was performed using the laparoscopic technique in 596 patients, the robot-assisted technique was used in 353 patients, whereas the transanal technique was performed in 249 patients. After excluding patients who underwent an APR, 344 patients remained in the laparoscopic group, 235 in the robot-assisted group, and 203 in the transanal group (Fig. 1). The majority of patients underwent surgery by the dedicated technique of the specific center. Patients in the TaTME centers were younger, had a lower tumor, and a shorter follow-up (Supplemental Table 1). In the TaTME centers, laparoscopic TME was used in 47 out of 90 patients who underwent an APR (48.0%), while laparoscopic TME was used in 15 out of 125 patients for APR in the R-TME centers (12.0%). Irrespective of the center, patients who underwent a TaTME were younger and had a lower tumor, and patients who underwent a TaTME or a R-TME less frequently had a history of abdominal surgery (Table 1).

**Table 1:** Baseline characteristics

	TME				LAR			
	Lap	Robot	TaTME	*P*	Lap	Robot	TaTME	*P*
	596	353	249		344	235	203	
Age (mean (SD)	68 (10)	67 (10)	65 (11)	0.001	68 (10)	66 (10)	64 (11)	0.001
BMI (mean (SD)	26 (4.1)	26 (4.0)	26 (4.5)	0.36	26 (4.2)	26 (3.9)	26 (4.3)	0.73
Sex (*n*, %)								
Male	377 (63.3)	228 (64.6)	165 (66.3)	0.70	213 (61.9)	150 (63.8)	136 (67.0)	0.49
Female	219 (36.7)	125 (35.4)	84 (33.7)		131 (38.1)	85 (36.2)	67 (33.0)	
ASA (*n*, %)								
I	109 (18.3)	74 (21.0)	53 (21.3)	0.17	70 (20.3)	49 (20.9)	47 (23.2)	0.22
II	359 (60.2)	202 (57.2)	152 (61.0)		197 (57.3)	145 (61.7)	127 (62.6)	
III	120 (20.1)	77 (21.8)	43 (17.3)		73 (21.2)	41 (17.4)	28 (13.8)	
IV	8 (1.3)	0 (0.0)	1 (0.4)		4 (1.2)	0 (0.0)	1 (0.5)	
History of abdominal surgery (%)								
No	403 (67.6)	266 (75.4)	185 (74.3)	0.02	230 (66.9)	180 (76.6)	154 (75.9)	0.01
Yes	193 (32.4)	87 (24.6)	64 (25.7)		114 (33.1)	55 (23.4)	49 (24.1)	
Distance to ARJ on MRI in cm (median [IQR])	5 [2, 8]	6 [3, 9]	4 [2, 6]	< 0.001	7 [5, 9]	8 [6, 9]	4 [3, 6]	< 0.001
Mesorectal fascia involvement (*n*, %)								
MRF +	189 (31.7)	119 (33.7)	78 (31.3)	0.83	73 (21.2)	66 (28.1)	61 (30.0)	0.08
MRF -	394 (66.1)	227 (64.3)	168 (67.5)		266 (77.3)	163 (69.4)	140 (69.0)	
Missing	13 (2.2)	7 (2.0)	3 (1.2)		5 (1.5)	6 (2.6)	2 (1.0)	
cT (*n*, %)								
1	13 (2.2)	5 (1.4)	10 (4.0)	0.12	11 (3.2)	5 (2.1)	9 (4.4)	0.48
2	167 (28.1)	110 (31.2)	69 (27.9)		95 (27.6)	74 (31.5)	52 (25.6)	
3	367 (61.7)	198 (56.1)	150 (60.7)		218 (63.4)	138 (58.7)	129 (63.5)	
4	48 (8.1)	40 (11.3)	18 (7.3)		20 (5.8)	18 (7.7)	12 (5.9)	
Missing	1 (0.2)	0 (0.0)	2 (0.8)		0 (0.0)	0 (0.0)	1 (0.5)	
cN (*n*, %)								
0	258 (43.4)	147 (41.6)	119 (47.8)	0.29	145 (42.2)	99 (42.1)	102 (50.2)	0.17
1	193 (32.5)	118 (33.4)	85 (34.1)		121 (35.2)	75 (31.9)	66 (32.5)	
2	143 (24.1)	88 (24.9)	45 (18.1)		76 (22.1)	61 (26.0)	35 (17.2)	
Missing	2 (0.3)	0 (0.0)	0 (0.0)		2 (0.6)	0 (0.0)	0 (0.0)	
cM (*n*, %)								
0	553 (93.1)	335 (95.4)	229 (92.3)	0.23	313 (91.0)	226 (96.2)	188 (92.6)	0.20
1	41 (6.9)	16 (4.6)	19 (7.7)		29 (8.4)	8 (3.4)	14 (6.9)	
Missing	2 (0.3)	2 (0.6)	1 (0.4)		2 (0.6)	1 (0.4)	1 (0.5)	
Neoadjuvant therapy (*n*, %)								
None	234 (39.7)	127 (36.2)	94 (37.8)	0.19	80 (23.3)	61 (26.0)	61 (30.0)	0.67
Chemoradiation	180 (30.5)	95 (27.1)	81 (32.5)		150 (43.6)	95 (40.4)	81 (39.9)	
Radiotherapie	176 (29.8)	129 (36.8)	74 (29.7)		113 (32.8)	78 (33.2)	61 (30.0)	
Missing	6 (1.0)	2 (0.6)	0 (0.0)		1 (0.3)	1 (0.4)	0 (0.0)	
Approach								
L-TME	596 (100.0)	0 (0.0)	0 (0.0)	< 0.001	344 (100.0)	0 (0.0)	0 (0.0)	< 0.001
R-TME	0 (0.0)	353 (100.0)	0 (0.0)		0 (0.0)	235 (100.0)	0 (0.0)	
TaTME	0 (0.0)	0 (0.0)	249 (100.0)		0 (0.0)	0 (0.0)	203 (100.0)	

*TME* total mesorectal excision, *LAR* low anterior resection, *Lap* laparoscopic, *Robot* robot-assisted, *TaTME* transanal TME, *P* *P*-value, *SD* standard deviation, *BMI* Body Mass Index, *ASA* American Society of Anesthesiology classification, *ARJ* anorectal junction, *MRI* magnetic resonance imaging, *IQR* interquartile range, *MRF* mesorectal fascia involvement, *cT* clinical T stage, *cN* clinical N stage, *cM* clinical M stage, *L-TME* laparoscopic TME, *R-TME* robot-assisted TME, *TaTME* transanal TME

### Stoma characteristics

In 4.9%, 8.5%, and 4.9% of the patients who underwent a LAR using the laparoscopic, robot-assisted, and transanal technique, respectively, a stoma was constructed prior to resection (*P* = 0.16). A significantly lower primary anastomosis rate was observed in patients who underwent a LAR using the laparoscopic technique compared to patients who underwent a LAR using the robot-assisted or transanal technique (72.4% versus 90.2% versus 88.2%, *P* < 0.001). After the initial resection, 70.9%, 72.3%, and 60.6% of the patients who underwent a LAR using the laparoscopic, robot-assisted, and transanal technique had a primary stoma, respectively (*P* < 0.001). Stoma construction due to a surgical complication, resulting in a secondary stoma, was observed in 7.3%, 5.1%, and 9.4% of the patients (*P* = 0.04). Construction of a new stoma after reversal (tertiary stoma) was performed in 4.9%, 6.4%, and 5.4% (*P* = 0.67) of the patients (Table 2).

**Table 2:** Stoma characteristic

	TME				LAR			
	Lap	Robot	TaTME	*P*	Lap	Robot	TaTME	*P*
	596	353	249		344	235	203	
Stoma before resection (*n*, %)	33 (5.5)	30 (8.5)	13 (5.2)	0.14	17 (4.9)	20 (8.5)	10 (4.9)	0.16
Diverting ileostomy	0 (0.0)	0 (0.0)	2 (15.4)	0.01	0 (0.0)	0 (0.0)	2 (20.0)	0.039
End ileostomy	0 (0.0)	0 (0.0)	1 (7.7)		0 (0.0)	0 (0.0)	1 (10.0)	
Diverting colostomy	21 (63.6)	21 (70.0)	8 (61.5)		14 (82.4)	18 (90.0)	7 (70.0)	
End colostomy	12 (36.4)	9 (30.0)	2 (15.4)		0 (0.0)	0 (0.0)	2 (20.0)	0.039
Primary anastomosis (*n*, %)	249 (41.8)	212 (60.1)	179 (71.9)	< 0.001	249 (72.4)	212 (90.2)	179 (88.2)	< 0.001
Reversal during resection (*n*, %)	6 (1.0)	5 (1.4)	3 (1.2)	0.85	4 (1.2)	3 (1.3)	3 (1.5)	0.95
Surgical procedure (*n*, %)								
APR	252 (42.3)	118 (33.4)	46 (18.5)*	< 0.001	0 (0.0)	0 (0.0)	0 (0.0)	< 0.001
LAR + anastomosis	100 (16.8)	65 (18.4)	80 (32.1)		100 (29.1)	65 (27.7)	80 (39.4)	
LAR + anastomosis + diverting stoma	149 (25.0)	147 (41.6)	99 (39.8)		149 (43.3)	147 (62.6)	99 (48.8)	
LAR + ostomy	95 (15.9)	23 (6.5)	24 (9.6)		95 (27.6)	23 (9.8)	24 (11.8)	
Primary stoma (*n*, %)	496 (83.2)	288 (81.6)	169 (67.9)*	< 0.001	244 (70.9)	170 (72.3)	123 (60.6) *	
Type of stoma after resection (*n*, %)								
Diverting ileostomy	123 (24.7)	135 (46.9)	94 (55.6)	< 0.001	123 (35.8)	135 (57.4)	94 (46.3)	< 0.001
End ileostomy	0 (0.0)	0 (0.0)	1 (0.6)		0 (0.0)	0 (0.0)	1 (0.5)	
Diverting colostomy	26 (5.2)	12 (4.1)	5 (3.0)		26 (7.6)	12 (5.1)	5 (2.5)	
End colostomy	347 (70.1)	141 (49.0)	69 (40.8)		95 (27.6)	23 (9.8)	23 (11.3)	
Surgical complications	201 (33.7)	116 (32.9)	82 (32.9)	0.95	115 (33.4)	91 (38.7)	66 (32.5)	0.31
Anastomotic leakage (*n*, %)	44 (17.7)	36 (17.0)	30 (16.8)	0.98	44 (17.7)	36 (17.0)	30 (16.8)	0.98
Reintervention (*n*, %)	101 (16.9)	53 (15.0)	55 (22.1)	0.07	68 (19.8)	39 (16.6)	46 (22.7)	0.28
Secondary stoma (*n*, %)	26 (4.4)	13 (3.7)	20 (8.0)	0.04	25 (7.3)	12 (5.1)	19 (9.4)	0.04
Deviating ileostomy	9 (34.6)	6 (46.2)	14 (70.0)	0.24	8 (32.0)	6 (50.0)	13 (68.4)	0.25
End ileostomy	1 (3.8)	1 (7.6)	0 (0.0)		1 (4.0)	0 (0.0)	0 (0.0)	
Deviating colostomy	7 (27.0)	4 (30.8)	2 (10.0)		7 (28.0)	4 (33.3)	2 (10.5)	
End colostomy	9 (34.6)	2 (15.4)	4 (20.0)		9 (36.0)	2 (16.7)	4 (21.1)	
Tertiary stoma (*n*, %)	17 (2.9)	16 (4.5)	11 (4.4)	0.65	17 (4.9)	15 (6.4)	11 (5.4)	0.67
Diverting ileostomy	8 (47.1)	7 (63.6)	7 (43.8)	0.74	8 (47.1)	7 (46.7)	7 (63.6)	0.78
End ileostomy	0 (0.0)	1 (9.1)	1 (6.2)		0 (0.0)	1 (6.7)	1 (9.1)	
Diverting colostomy	2 (11.8)	1 (9.1)	1 (6.2)		2 (11.8)	1 (6.7)	1 (9.1)	
End colostomy	7 (41.2)	2 (18.2)	7 (43.8)		7 (41.2)	6 (40.0)	2 (18.2)	
Functional anastomosis (*n*, %)								
1 year	210 (35.2)*	182 (51.6)	152 (61.0)	< 0.001	210 (61.0)*	182 (77.4)	152 (74.9)	< 0.001
3 year	206 (34.6)*	187 (53.0)	149 (59.8)	< 0.001	206 (59.9)*	187 (79.6)	149 (73.4)	< 0.001
Permanent stoma (end of FU) (*n*, %)	390 (65.4)*	166 (47.0)	97 (39.0)	< 0.001	138 (40.1)*	50 (21.3)	52 (25.6)	< 0.001
Follow up in months (median, [IQR])	38 [28, 50]	37 [27, 47]	35 [26, 47]	0.02	37 [27, 49]	38 [27, 47]	36 [26, 46]	0.39

*TME* total mesorectal excision, *LAR* low anterior resection, *Lap* laparoscopic, *Robot* robot-assisted, *TaTME* transanal TME, *P* *P* value, *APR* abdominoperineal resection, *FU* follow-up, *FU* follow-up, *IQR* interquartile range.*Significant after post hoc testing

### Permanent stoma rate

A permanent stoma at the end of follow-up in patients who underwent a LAR was observed in 40.1%, 21.3%, and 25.6% of the patients in the laparoscopic, robot-assisted, and TaTME group, respectively (*P* < 0.001). For patients undergoing a restorative LAR, this was 17.3%, 11.8%, and 15.1% (*P* = 0.26), respectively (Tables 2 and 3). Reversal of a primary stoma in patients who underwent a restorative LAR was performed in 88.6%, 94.6%,and 87.9% of the laparoscopic, robot-assisted and transanal group (*P* = 0.19) (Table 3). Multivariable regression analysis of patients undergoing a LAR using the robot-assisted technique (OR 0.39 [95% CI 0.25, 0.59]) and transanal technique (OR 0.35 [95% CI 0.22, 0.55]) was associated with a lower permanent stoma rate compared to the laparoscopic technique. (Table 4) Other variables independently associated with permanent stoma rate were age, history of abdominal surgery, ASA classification, distance to the ARJ, and neoadjuvant therapy. In patients undergoing a restorative LAR, the multivariable regression analysis showed that R-TME and TaTME were not associated with permanent stoma rate. Variables independently associated with permanent stoma rate were history of abdominal surgery, ASA classification, distance to the ARJ, anastomotic leakage, and the construction of a diverting stoma during initial resection. (Table 4).

**Table 3:** Reversal of diverting stoma and functional anastomosis rate in patients undergoing a restorative LAR

	Restorative LAR			*P*
	Lap	Robot	TaTME	
	249	212	179	
Stoma reversal of primary stoma (*n*, %)	132 (88.6)	139 (94.6)	87 (87.9)	0.19
Time to reversal in days (median [IQR])	96 [69, 150]	108 [88, 150]	107 [78, 180]	0.05
Stoma reversal of secondary stoma (*n*, %)	9 (34.6)	6 (46.2)	13 (65.0)	0.29
Time to reversal in days (median [IQR])	143 [131, 175]	414 [227, 465]	191 [139, 273]	0.12
Functional anastomosis (*n*, %)				
1 year	210 (84.3)	182 (85.8)	152 (84.9)	0.90
3 year	206 (82.7)	187 (88.2)	152 (84.9)	0.26
Permanent stoma (end of FU) (*n*, %)	43 (17.3)	25 (11.8)	27 (15.1)	0.26

*TME* total mesorectal excision, *LAR* low anterior resection, *Lap* laparoscopic, *Robot* robot-assisted, *TaTME* transanal TME, *IQR* interquartile range, *FU* follow-up. * Significant after post-hoc testing

**Table 4:** Multivariable regression analyses for permanent stoma rate at the end of follow in all TME patients versus in all low anterior resection patients (with or without the construction of a ending colostomy) versus in patients undergoing a restorative low anterior resection

	TME		LAR		Restorative LAR	
	OR (95% CI)	*P*	OR (95% CI)	*P*	OR (95% CI)	*P*
	Reference		Reference		Reference	
Technique						
Lap						
Robot	0.48 [0.34, 0.68]	< 0.001	0.39 [0.25, 0.59]	< 0.001		
TaTME	0.17 [0.11, 0.25]	< 0.001	0.35 [0.22, 0.55]	< 0.001		
Age	1.07 [1.05, 1.09]	< 0.001	1.08 [1.06, 1.10]	< 0.001		
BMI	1.05 [1.01, 1.09]	0.005	1.04 [0.99, 1.08]	0.07		
Sex						
Female	0.74 [0.54, 1.02]	0.07			0.65 [0.37, 1.12]	0.13
History of abdominal surgery	1.47 [1.05, 2.05]	0.03	1.49 [1.03, 2.17]	0.03	1.84 [1.03, 3.22]	0.04
ASA						
II						
I	1.13 [0.77, 1.70]	0.54	1.24 [0.76, 2.00]	0.38	1.78 [0.94, 3.31]	0.07
III	2.54 [1.68, 3.85]	< 0.001	2.05 [1.31, 3.21]	0.002	2.72 [1.34, 5.43]	0.005
IV	1.32 [0.24, 8.39]	0.76	0.99 [0.12, 6.54]	1.00	3.24 [0.15, 30.7]	0.34
Distance to ARJ on MRI in cm	0.65 [0.61, 0.69]	< 0.001	0.83 [0.77, 0.89]	< 0.001	0.90 [0.82, 0.98]	0.02
No MRF involvement	0.55 [0.38, 0.79]	0.001	0.72 [0.46, 1.11]	0.13		
cM1					2.11 [0.90, 4.73]	0.08
Neoadjuvant therapy						
None						
Radiotherapy	1.29 [0.91, 1.84]	0.15	1.39 [0.92, 2.10]	0.11		
Chemoradation	1.70 [1.27, 2.56]	0.01	1.85 [1.14, 3.00]	0.01		
Length of follow-up (months)	0.99 [0.98, 1.00]	0.08	0.98 [0.97, 0.99]	0.006	0.98 [0.96, 1.00]	0.06
Anastomotic leakage					10.66 [6.27, 18.4]	< 0.001
Diverting stoma					2.68 [1.53, 4.88]	< 0.001

*OR* odds ratio, *CI* confidence interval, *P* *P*-value, *TME* total mesorectal excision, *LAR* low anterior resection, *Lap* laparoscopic, *Robot* robot-assisted, *TaTME* transanal TME, *BMI* body mass index, *ASA* American Society of Anesthesiology, *ARJ* anorectal junction, *MRI* magnetic resonance imaging, *MRF* mesorectal fascia, *cM* clinical M stage

### Stoma-related complications

Stoma complications were present in 39.1% of the patients with a diverting ileostomy, 66.7% of the patients with an end ileostomy, 44.1% of the patients with a diverting colostomy, and 49.6% of the patients with an end colostomy. Stoma complications within 30 days after the initial resection were mostly seen in patients with a diverting ileostomy. High-output stoma and ileus were most frequently seen in this group, while necrosis was most frequently seen in patients with an end colostomy. Stoma complications after 30 days were mostly seen in patients with an end colostomy, with parastomal hernia being the most frequent complication. Skin complications were seen in all four types of stomas. (Table 5) Multivariable regression analysis showed that years of having a stoma was independently associated with the occurrence of overall stoma complications (OR 1.29 [95% CI 1.19, 1.40]).

**Table 5:** Stoma complications stratified by type of stoma

	Total	Diverting ileostomy	End ileostomy	Diverting colostomy	End colostomy	*P*
	1003	372	3	59	569	
Total stoma complications (*n*, %)	455 (45.4)	145 (39.1)	2 (66.7)	26 (44.1)	282 (49.6)	0.01
Complications < 30 days (*n*, %)	134 (13.4)	93 (25.0)	0 (0.0)	7 (11.9)	34 (6.0)	< 0.001
Ileus	24 (17.9)	17 (18.3)	0 (0.0)	2 (28.6)	5 (14.7)	
High output	76 (56.7)	70 (75.3)	0 (0.0)	2 (28.6)	4 (11.8)	
Prolaps	6 (4.5)	1 (1.1)	0 (0.0)	3 (42.9)	2 (5.9)	
Parastomal hernia	5 (3.7)	3 (3.2)	0 (0.0)	0 (0.0)	2 (5.9)	
Dehiscence	5 (3.7)	3 (3.2)	0 (0.0)	0 (0.0)	2 (5.9)	
Necrosis	14 (10.4)	0 (0.0)	0 (0.0)	1 (14.3)	13 (38.2)	
Infection	5 (3.7)	1 (1.1)	0 (0.0)	1 (14.3)	3 (8.8)	
Skin complications	12 (3.2)	4 (4.3)	0 (0.0)	0 (0.0)	1 (2.9)	
Other	5 (3.7)	1 (1.1)	0 (0.0)	1 (14.3)	3 (8.8)	
Complications > 30 days (*n*, %)	370 (37.0)	81 (22.0)	2 (66.7)	24 (40.7)	263 (46.2)	< 0.001
Ileus	12 (3.2)	6 (7.4)	0 (0.0)	1 (4.2)	5 (1.9)	
High output	23 (6.2)	18 (22.2)	0 (0.0)	1 (4.2)	4 (1.5)	
Prolaps	52 (14.1)	9 (11.1)	0 (0.0)	18 (75.0)	25 (9.5)	
Parastomal hernia	190 (51.4)	7 (8.6)	1 (50.0)	7 (29.2)	175 (66.5)	
Dehiscence	5 (1.4)	1 (1.2)	0 (0.0)	0 (0.0)	4 (1.5)	
Necrosis	8 (2.2)	3 (3.7)	0 (0.0)	2 (8.3)	3 (1.1)	
Infection	3 (0.8)	0 (0.0)	0 (0.0)	1 (4.2)	2 (0.8)	
Skin complications	172 (46.5)	51 (63.0)	1 (50.0)	7 (29.2)	113 (43.0)	
Stricture	8 (2.2)	2 (2.5)	0 (0.0)	1 (4.2)	5 (1.9)	
Other	12 (3.2)	1 (1.2)	0 (0.0)	2 (8.3)	9 (3.4)	
Duration of stoma in months (median [IQR])	27 [4,41]	4 [3, 7]	44 [40, 48]	5 [3, 22]	36 [27, 49]	< 0.001

*P* *P* value, *n* number of patients, *IQR* interquartile range

### Stoma-related readmissions and reoperations

40 (4.0%) patients experienced one or more readmissions during the follow-up, with the majority of the patients (82.5%) being readmitted only once. Additionally, 89 patients (8.9%) underwent one or more reoperations. (Table 6) Multivariable regression analysis showed that a diverting colostomy (OR 4.83 [95% CI 1.88, 12.03]) and years of having a stoma (OR 1.83 [95% CI 1.52, 2.20]) were independently associated with stoma-related reoperations.

**Table 6:** Stoma-related readmissions and surgical interventions

	Overall
Patients who had/have a stoma	1003
Readmission (*n*, %)	40 (4.0)
1 readmission	33 (82.5)
2 readmissions	6 (15.0)
3 or more readmissions	1 (2.5)
Length of stay of readmission (median [IQR])	
1st readmission	4 [2, 8]
2nd readmission	9 [6, 10]
3rd readmission	2 [2]
Reoperation (*n*, %)	89 (8.9)
1 intervention	73 (82.0)
2 interventions	12 (13.5)
3 or more interventions	4 (4.5)
Type of reoperation (*n*, %)	
Local revision	35
Diverting to end ostomy	41
Sugarbaker	8
Other	24

*n* number, *IQR* interquartile range

## Discussion

This study aimed to evaluate permanent stoma rate in patients undergoing minimally invasive rectal cancer surgery. In this study, patients undergoing a LAR using the robot-assisted or transanal technique were independently associated with a lower permanent stoma rate compared to the laparoscopic technique. Additionally, a high rate of stoma-related complications, readmissions, and reoperations was observed.

Previous studies already suggested an increased anastomosis rate using the transanal and robot-assisted technique, without an increase in anastomotic leakage rate [14, 15, 17]. However, it was yet unclear whether this would also result in a lower permanent stoma rate. In this study, the robot-assisted and transanal techniques were independently associated with a lower permanent stoma rate in the total group of patients who underwent a TME, and in the group of patients who underwent a LAR. In patients who underwent a restorative LAR, these minimally invasive techniques were not associated with permanent stoma rate. This strengthens the hypothesis that the lower permanent stoma rate might be due to higher anastomosis rate using the robot-assisted and transanal techniques. Additionally, this suggests that the increased anastomotic rate using the robot-assisted and transanal techniques does not come with a higher anastomotic break-down during 3-year follow-up, resulting in a lower permanent stoma rate. The improved visibility in both techniques might explain the increase in primary anastomosis rate, and the subsequent associated lower permanent stoma rate. Perhaps, with enhanced visibility, stapling could be easier: the robot-assisted technique comes with more degrees of freedom, thereby potentially reducing stapling difficulties. The effect on permanent stoma rate was stronger in the transanal group (OR 0.17) than the robot-assisted group (OR 0.48) for all TME patients, while the effect was comparable between the transanal group (OR 0.35) and the robot-assisted group (OR 0.39) in patients who underwent a LAR. Probably this is because TaTME is mostly not used for an APR, while the laparoscopic approach is used in these cases. This causes selection bias, and therefore, the association of TaTME with permanent stoma rate will probably be lower. Nevertheless, after excluding patients with an APR, the association of the robot-assisted and transanal techniques with permanent stoma rate remains, supporting or hypothesis that the robot-assisted and transanal techniques are associated with a higher primary anastomosis rate and a lower permanent stoma rate.

Variables associated with permanent stoma rate other than the technique were age, history of abdominal surgery, ASA classification, lower distance to the ARJ, chemoradiation, anastomotic leakage, and diverting stoma during initial resection. Two recent large nation-wide studies showed comparable risk factors for permanent stoma rate. However, minimally invasive surgery was not associated with a reduced permanent stoma rate [30, 31], perhaps since they did not separately identify R-TME, L-TME, and TaTME. Or this is due to the fact that the current study only included patients operated in dedicated centers.

This study shows a significant rate of stoma-related complications: 45.4% of the patients experienced stoma-related complications, 4.0% of the patients were at least once readmitted, and 8.9% of the patients underwent at least one reoperation, irrespective of admission and reoperation related to reversal of a diverting stoma. This is in line with other studies showing considerable stoma-related complications, readmissions, and reoperations [32–34]. Despite the presented rates, stoma-related morbidity with a follow-up duration of only 36 months still might underestimate the actual morbidity, especially in permanent stomas, as the incidence of complications increases with the duration of having a stoma.

Although the robot-assisted and transanal techniques were associated with a lower permanent stoma rate, it is unclear how this will affect quality of life [35, 36]. It is suggested that quality of life might be better in patients with a restorative resection [9, 10]. However, some studies show worse quality of life in patients with restorative resections compared to patients with a permanent stomay [37, 38]. This might be especially important for patients with a low rectal tumor, as the risk of low anterior resection syndrome is higher in these patients receiving a primary anastomosis. Additionally, the reduction in permanent stoma rate may also have an effect on healthcare costs, as costs associated with daily care of the stoma, stoma-related complications, readmissions, and reinterventions occur more often in patients having a permanent stoma. Both aspects should be taken into account in future analyses.

Certain limitations should be taken into account when interpreting the results. First, this is a retrospective study, hence bias might be present. We tried to control for confounding by indication by performing a multivariable regression analysis, although residual confounding might still be present. Furthermore, TaTME is generally not used to perform an APR, both rather using the laparoscopic technique. As an effect permanent stoma rate could be underestimated, whereas primary anastomosis rate and functional anastomosis rate could be overestimated. To account for this, we excluded the patients undergoing an APR from the primary outcome. However, by excluding these patients from all the analyses, selection bias might be present as well. As patients with a low tumor might be offered a restorative procedure in a robot-assisted or TaTME center, this would not have been offered in a laparoscopic center, as the robot-assisted and transanal techniques are suggested to provide better overview in low rectal tumors, thereby enabling sphincter-saving surgery in more patients. Therefore, we presented data regarding all TME patients, LAR patients, and restorative LAR patients, stratified per technique (laparoscopic, robot-assisted, and transanal) and stratified per center (laparoscopic center, robot-assisted center, and transanal centers). Furthermore, stoma complications might be underestimated due to the retrospective nature of the study. Nevertheless, permanent stoma rate, readmissions, and reinterventions are generally well documented, and less prone to report bias.

Second, the difference in permanent stoma rate, which might be caused by difference in primary anastomosis rate, might not only be associated with the technique: surgeon-related or center-related preferences might play a part as well. Unfortunately, controlling for surgeons was not possible as we did not register this. Furthermore, controlling for centers in the multivariable regression analysis was not possible, as this would lead to multicollinearity. However, no large differences of the crude permanent stoma rates within L-TME, R-TME, and TaTME centers were observed.

Third, the present cohort consists of patients operated by experienced surgeons. Therefore, these results might not directly be extrapolated to all rectal cancer patients. Finally, we used the MRI-based definition for rectal cancer as proposed by D’Souza et al., thereby rectosigmoid tumors that might have been included if former definitions were used were excluded from this analysis. The inclusion of a relatively higher proportion of patients with a low rectal tumor could have led to a relatively high permanent stoma rate.

As this is the first study showing an association between surgical approach and permanent stoma rate, future prospective studies are necessary to confirm our results. Additionally, the impact on quality of life and costs should be investigated. Especially in low rectal tumors, more prospective data are necessary to assess risk of low anterior resection syndrome and its associated influence on quality of life and cost-effectivity in case of restorative rectal resection.

In conclusion, the robot-assisted and TaTME technique are associated with a lower permanent stoma rate compared to the laparoscopic technique in patients undergoing a LAR. This association might be an effect of the higher primary anastomosis rate in these two techniques.

### Supplementary Information

Below is the link to the electronic supplementary material.

Supplementary file1 (DOCX 27 kb)

Supplementary file2 (DOCX 26 kb)

Supplementary file3 (DOCX 18 kb)

## Data Availability

Data are available on reasonable request, this includes template data collection forms, data extracted from included studies, data used for analyses, and any other materials used in the review.

## Code availability

Code is available on request.
