# Supplementary material for: Permanent stoma rate and long-term stoma complications in laparoscopic, robot-assisted, and transanal total mesorectal excisions: a retrospective cohort study
Source: Surg Endosc. 2023 Nov 6;38(1):105–15. doi: 10.1007/s00464-023-10517-9 (PMC10776460; doi:10.1007/s00464-023-10517-9)
Supplement: Supplementary file 1 — Supplementary file1 (DOCX 27 kb) [file 464_2023_10517_MOESM1_ESM.docx]

|  |  | TME | | |  |  | LAR | | |  |
| --- | --- | --- | --- | --- | --- | --- | --- | --- | --- | --- |
|  |  | Lap centre | Robot centre | TaTME centre | p |  | Lap centre | Robot centre | TaTME centre | p |
|  |  | 502 | 397 | 299 |  |  | 301 | 272 | 209 |  |
| Age (mean (SD)) |  | 68 (10) | 67 (10) | 65 (11) | 0.006 |  | 68 (9) | 66 (10) | 64 (11) | 0.001 |
| BMI (mean (SD)) |  | 26 (4.3) | 26 (4.0) | 26 (4.1) | 0.64 |  | 26 (4.6) | 2(3.9) | 26 (3.8) | 0.50 |
| Sex (n, %) | Male | 321 (63.9) | 251 (63.2) | 198 (66.2) | 0.70 |  | 186 (61.8) | 167 (61.4) | 146 (69.9) | 0.10 |
|  | Female | 181 (36.1) | 146 (36.8) | 101 (33.8) |  |  | 115 (38.2) | 105 (38.6)6 | 63 (30.1) |  |
| ASA (n, %) | I | 97 (19.3) | 81 (20.4) | 58 (19.4) | 0.09 |  | 66 (21.9) | 55 (20.2) | 45 (21.5) | 0.13 |
|  | II | 298 (59.4) | 228 (57.4) | 187 (62.5) |  |  | 168 (55.8) | 166 (61.0) | 135 (64.6) |  |
|  | III | 99 (19.7) | 88 (22.2) | 53 (17.7) |  |  | 63 (20.9) | 51 (18.8) | 28 (13.4) |  |
|  | IV | 8 (1.6) | 0 (0.0) | 1 (0.3) |  |  | 4 (1.3) | 0 (0.0) | 1 (0.5) |  |
| History of abdominal surgery (%) | No | 346 (68.9) | 296 (74.6) | 212 (70.9) | 0.18 |  | 203 (67.4) | 204 (75.0) | 157 (75.1) | 0.07 |
|  | Yes | 156 (31.1) | 101 (25.4) | 87 (29.1) |  |  | 98 (32.6) | 68 (25.0) | 52 (24.9) |  |
| Distance to ARJ on MRI in cm (median [IQR]) |  | 5 [2, 8] | 6 [3, 9] | 4 [1, 6] | <0.001 |  | 7 [5, 9] | 8 [6, 9] | 5 [3, 6] | <0.001 |
| Mesorectal fascia involvement (n, %) | MRF + | 151 (30.1) | 129 (32.5) | 106 (35.5) | 0.61 |  | 60 (19.9) | 76 (27.9) | 64 (30.6) | 0.026 |
|  | MRF - | 340 (67.7) | 261 (65.7) | 188 (62.9) |  |  | 237 (78.7) | 189 (69.5) | 143 (68.4) |  |
|  | Missing | 11 (2.2) | 7 (1.8) | 5 (1.7) |  |  | 4 (1.3) | 7 (2.6) | 2 (1.0) |  |
| cT (n, %) | 1 | 9 (1.8) | 9 (2.3) | 10 (3.3) | 0.23 |  | 8 (2.7) | 8 (2.9) | 9 (4.3) | 0.47 |
|  | 2 | 147 (29.3) | 119 (30.0) | 80 (26.8) |  |  | 90 (29.9) | 79 (29.0) | 52 (25.0) |  |
|  | 3 | 310 (61.8) | 226 (56.9) | 179 (59.9) |  |  | 189 (62.8) | 164 (60.3) | 132 (63.5) |  |
|  | 4 | 35 (7.0) | 43 (10.8) | 28 (9.4) |  |  | 14 (4.7) | 21 (7.7) | 15 (7.2) |  |
|  | Missing | 1 (0.2) | 0 (0.0) | 2 (0.7) |  |  | 0 (0.0) | 0 (0.0) | 1 (0.5) |  |
| cN (n, %) | 0 | 217 (43.2) | 162 (40.8) | 145 (48.5) | 0.31 |  | 128 (42.7) | 113 (41.5) | 105 (50.5) | 0.12 |
|  | 1 | 168 (33.5) | 132 (33.2) | 96 (32.1) |  |  | 103 (34.3) | 88 (32.4) | 71 (34.1) |  |
|  | 2 | 116 (23.1) | 103 (25.9) | 57 (19.1) |  |  | 69 (23.0) | 71 (26.1) | 32 (15.4) |  |
|  | Missing | 1 (0.2) | 0 (0.0) | 1 (0.3) |  |  | 1 (0.3) | 0 (0.0) | 1 (0.5) |  |
| cM (n, %) | 0 | 473 (94.2) | 373 (94.0) | 271 (90.6) | 0.11 |  | 280 (93.0) | 257 (95.2) | 190 (91.8) | 0.29 |
|  | 1 | 29 (5.8) | 21 (5.3) | 26 (8.7) |  |  | 21 (7.0) | 13 (4.8) | 17 (8.2) |  |
|  | Missing | 0 (0.0) | 3 (0.8) | 2 (0.7) |  |  | 0 (0.0) | 2 (0.7) | 2 (1.0) |  |
| Neoadjuvant therapy (n, %) | None | 198 (39.4) | 144 (36.3) | 113 (37.8) | 0.14 |  | 131 (43.7) | 108 (39.9) | 87 (41.6) | 0.86 |
|  | Chemoradiation | 149 (29.7) | 106 (26.7) | 101 (33.8) |  |  | 73 (24.3) | 70 (25.8) | 59 (28.2) |  |
|  | Radiotherapie | 150 (29.9) | 145 (36.5) | 84 (28.1) |  |  | 96 (32.0) | 93 (34.3) | 63 (30.1) |  |
|  | Missing | 5 (1.0) | 2 (0.5) | 1 (0.3) |  |  | 1 (0.3) | 1 (0.4) | 0(0.0) |  |
| Approach (n, %) | L-TME | 468 (93.2) | 55 (13.9) | 73 (24.4) | <0.001 |  | 278 (92.4) | 40 (14.7) | 26 (12.4) | <0.001 |
|  | R-TME | 21 (4.2) | 2 (0.5) | 226 (75.6) |  |  | 18 (6.0) | 2 (0.7) | 183 (87.6) |  |
|  | TaTME | 13 (2.6) | 340 (85.6) | 0 (0.0) |  |  | 5 (1.7) | 230 (84.6) | 0 (0.0) |  |

**Table 1:** Baseline characteristics of patients stratified per technique: laparoscopic, robot-assisted and transanal. TME: total mesorectal excision, LAR: low anterior resection, Lap: laparoscopic, Robot: robot-assisted, TaTME: transanal TME, p: p-value, SD: standard deviation, BMI: Body Mass Index, ASA: American Society of Anesthesiology classification, ARJ: anorectal junction, MRI: magnetic resonance imaging, IQR: interquartile range, MRF: mesorectal fascia involvement, cT: clinical T stage, cN: clinical N stage, cM: clinical M stage, L-TME: laparoscopic TME, R-TME: robot-assisted TME, TaTME: transanal TME.
