# Supplementary material for: Permanent stoma rate and long-term stoma complications in laparoscopic, robot-assisted, and transanal total mesorectal excisions: a retrospective cohort study
Source: Surg Endosc. 2023 Nov 6;38(1):105–15. doi: 10.1007/s00464-023-10517-9 (PMC10776460; doi:10.1007/s00464-023-10517-9)
Supplement: Supplementary file 2 — Supplementary file2 (DOCX 26 kb) [file 464_2023_10517_MOESM2_ESM.docx]

|  |  | TME | | |  |  | LAR | | |  |
| --- | --- | --- | --- | --- | --- | --- | --- | --- | --- | --- |
|  |  | Lap centre | Robot centre | TaTME centre | p |  | Lap centre | Robot centre | TaTME centre | p |
|  |  | 502 | 397 | 299 |  |  | 301 | 272 | 209 |  |
| Stoma before resection (n, %) |  | 28 (5.6) | 33 (8.3) | 15 (5.0) | 0.14 |  | 14 (4.7) | 24 (8.8) | 9 (4.3) | 0.05 |
|  | Diverting ileostomy | 0 (0.0) | 0 (0.0) | 2 (13.3) | 0.03 |  | 0 (0.0) | 0 (0.0) | 2 (22.2) |  |
|  | End ileostomy | 0 (0.0) | 0 (0.0) | 1 (6.7) |  |  | 0 (0.0) | 0 (0.0) | 1 (11.1) |  |
|  | Deviating colostomy | 18 (64.3) | 25 (75.8) | 7 (46.7) |  |  | 12 (85.7) | 22 (91.7) | 5 (55.6) |  |
|  | End colostomy | 10 (35.7) | 8 (24.2) | 5 (33.3) |  |  | 2 (14.3) | 2 (8.3) | 1 (11.1) |  |
| Primary anastomosis (n, %) |  | 207 (41.2)* | 243 (61.2) | 190 (63.5) | <0.001 |  | 207 (68.8)* | 243 (89.3) | 190 (90.9) | <0.001 |
| Reversal during resection (n, %) |  | 5 (1.0) | 6 (1.5) | 3 (1.0) | 0.74 |  | 3 (1.0) | 4 (1.5) | 3 (1.4) | 0.86 |
| Surgical procedure (n, %) | APR | 201 (40.0) | 125 (31.5) | 90 (30.1) | <0.001 |  | 0 (0.0) | 0 (0.0) | 0 (0.0) | <0.001 |
|  | LAR + anastomosis | 85 (16.9) | 69 (17.4) | 91 (30.4) |  |  | 85 (28.2) | 69 (25.4) | 91 (43.5) |  |
|  | LAR + anastomosis + Diverting stoma | 122 (24.3) | 174 (43.8) | 99 (33.1) |  |  | 122 (40.5) | 174 (64.0) | 99 (47.4) |  |
|  | LAR + ostomy | 94 (18.7) | 29 (7.3) | 19 (6.4) |  |  | 94 (31.2) | 29 (10.7) | 19 (9.1) |  |
| Primary stoma (n, %) |  | 417 (83.1) | 328 (82.6) | 208 (69.8) | <0.001 |  | 216 (71.8) | 203 (74.6) | 118 (56.5) | <0.001 |
| Type of stoma after resection (n, %) | Diverting ileostomy | 96 (23.0) | 158 (48.1) | 98 (47.1) | <0.001 |  | 96 (31.9) | 158 (58.1) | 98 (46.9) | <0.001 |
|  | End ileostomy | 0 (0.0) | 0 (0.0) | 1 (0.5) |  |  | 0 (0.0) | 0 (0.0) | 1 (0.5) |  |
|  | Diverting colostomy | 26 (6.2) | 16 (4.9) | 1 (0.5) |  |  | 26 (8.6) | 16 (5.9) | 1 (0.5) |  |
|  | End colostomy | 295 (70.8) | 154 (47.0) | 108 (51.9) |  |  | 94 (31.2) | 29 (10.7) | 18 (8.6) |  |
| Surgical complications |  | 176 (35.1) | 133 (33.5) | 90 (30.1) | 0.35 |  | 106 (35.2) | 107 (39.3) | 59 (28.2) | 0.04 |
| Anastomotic leakage (n, %) |  | 36 (17.6) | 42 (17.4) | 32 (16.8) | 0.98 |  | 36 (17.6) | 42 (17.4) | 32 (16.8) | 0.98 |
| Reintervention (n, %) |  | 94 (18.7) | 61 (15.4) | 54 (18.1) | 0.40 |  | 64 (21.3) | 46 (16.9) | 43 (20.6) | 0.39 |
| Secondary stoma (n, %) |  | 22 (4.4) | 14 (3.5) | 23 (7.7) | 0.04 |  | 21 (7.0) | 13 (4.8) | 22 (10.5) | 0.03 |
|  | Diverting ileostomy | 6 (27.3) | 6 (42.9) | 17 (73.9) | 0.04 |  | 5 (23.8) | 6 (46.2) | 16 (72.7) | 0.03 |
|  | End ileostomy | 1 (4.5) | 1 (7.1) | 0 (0.0) |  |  | 1 (4.8) | 0 (0.0) | 0 (0.0) |  |
|  | Diverting colostomy | 6 (27.3) | 5 (35.7) | 2 (8.7) |  |  | 6 (28.6) | 5 (38.5) | 2 (9.1) |  |
|  | End colostomy | 9 (40.9) | 2 (14.3) | 4 (17.4) |  |  | 9 (42.9) | 2 (15.4) | 4 (18.2) |  |
| Tertiary stoma (n, %) |  | 15 (3.0) | 18 (4.5) | 11 (3.4) | 0.80 |  | 15 (5.0) | 17 (6.3) | 11 (5.3) |  |
|  | Diverting ileostomy | 8 (53.3) | 6 (33.3) | 8 (72.7) | 0.32 |  | 8 (53.3) | 6 (35.3) | 8 (72.7) | 0.35 |
|  | End ileostomy | 0 (0.0) | 1 (5.6) | 1 (9.1) |  |  | 0 (0.0) | 1 (5.9) | 1 (9.1) |  |
|  | Diverting colostomy | 1 (6.7) | 3 (16.7) | 0 (0.0) |  |  | 1 (6.7) | 3 (17.6) | 0 (0.0) |  |
|  | End colostomy | 6 (40.0) | 8 (44.4) | 2 (18.2) |  |  | 6 (40.0) | 7 (41.2) | 2 (18.2) |  |
| Functional anastomosis (n, %) | 1 year | 175 (34.9)* | 206 (51.9) | 163 (54.5) | <0.001 |  | 175 (58.1)* | 206 (75.7) | 163 (78.0) | <0.001 |
|  | 3 year | 172 (34.3)* | 201 (50.6) | 160 (53.5) | <0.001 |  | 172 (57.1)* | 201 (73.9) | 160 (0.77) | <0.001 |
| Permanent stoma (end of FU) (n, %) |  | 331 (65.9) | 187 (47.1) | 138 (46.2) | <0.001 |  | 130 (43.2) | 62 (22.8) | 48 (23.0) | <0.001 |
| Follow-up in months (median [IQR]) |  | 37 [29, 49] | 38 [27, 48] | 35 [25, 48] | 0.03 |  | 37 [28, 48] | 38 [27, 48] | 36 [26, 47] | 0.19 |

**Supplemental table 2:** Stoma characteristics. TME: total mesorectal excision, LAR: low anterior resection, Lap: laparoscopic, Robot: robot-assisted, TaTME: transanal TME, p: p-value, APR: abdominoperineal resection, FU: follow-up, IQR: interquartile range.* Significant after post-hoc testing.
