# Supplementary material for: Permanent stoma rate and long-term stoma complications in laparoscopic, robot-assisted, and transanal total mesorectal excisions: a retrospective cohort study
Source: Surg Endosc. 2023 Nov 6;38(1):105–15. doi: 10.1007/s00464-023-10517-9 (PMC10776460; doi:10.1007/s00464-023-10517-9)
Supplement: Supplementary file 3 — Supplementary file3 (DOCX 18 kb) [file 464_2023_10517_MOESM3_ESM.docx]

|  |  |  | Restorative LAR | | |  |
| --- | --- | --- | --- | --- | --- | --- |
|  |  |  | L-TME  centre | R-TME  centre | TaTME  centre | p |
|  |  |  | 207 | 243 | 190 |  |
| Stoma reversal of primary stoma (n, %) | |  | 108 (88.5) | 163 (93.7) | 87 (87.9) | 0.22 |
| Time to reversal (median [IQR]) | |  | 94 [64, 154] | 106 [88, 147] | 107 [77, 178] | 0.07 |
| Stoma reversal of secondary stoma (n, %) | |  | 9 (34.6) | 6 (46.2) | 13 (65.0) | 0.29 |
| Time to reversal (median [IQR]) | |  | 143 [131, 175] | 414 [227, 465] | 191 [139, 273] | 0.12 |
| Permanent stoma (end of FU) (n, %) |  |  | 36 (17.4) | 33 (13.6) | 29 (15.3) | 0.54 |
| Functional anastomosis (n, %) | 1 year |  | 175 (84.5) | 206 (84.8) | 163 (85.8) | 0.93 |
|  | 3 year |  | 172 (83.1) | 201 (82.7) | 160 (84.2) | 0.91 |

**Supplemental table 3:** Reversal of diverting stoma and functional anastomosis rate in patients undergoing a restorative LAR. TME: total mesorectal excision, LAR: low anterior resection, Lap: laparoscopic, Robot: robot-assisted, TaTME: transanal total mesorectal excision, IQR: interquartile range, FU: follow up. * Significant after post-hoc testing.
